# Driver, vehicle, and environmental risk factors for crash-level major trauma in drunk driving collisions: a national registry linkage study

**DOI:** 10.3389/fpubh.2026.1733604

**Published:** 2026-09-28

**Authors:** Ling-Wei Kuo, Chien-An Liao, Chi-Tung Cheng, Chien-Hung Liao, Chih-Po Hsu, Chih-Yuan Fu, Chun-Shiang Yang, Jen-Fu Huang

**Affiliations:** 1 Department of Trauma and Emergency Surgery, Chang Gung Memorial Hospital, Linkou Medical Center, Taoyuan City, Taiwan; 2 Institute of Biomedical Engineering, College of Medicine and College of Engineering, National Taiwan University, Taipei City, Taiwan; 3 New Taipei City Police Department, New Taipei City, Taiwan; 4 Division of Trauma and Emergency Surgery, Department of General Surgery, Jen-Ai Hospital, Dali Branch, Taichung, Taiwan

**Keywords:** alcohol, drunk-driving, major trauma, risk assessment, traffic crash

## Abstract

**Introduction:**

Alcohol can impair driving performance, and drunk driving is associated with traffic crashes and injuries. However, there is a lack of evidence regarding the driver-related, vehicle-related and environmental risk factors for major trauma in drunk driving crashes.

**Methods:**

We retrospectively analyzed crash-level data from the National Health Insurance Research Database (NHIRD) and Traffic Accident Data (TAD) from 2012 to 2019. Data from all the drunk-driving violations from this period were extracted, and we categorized these violations based on whether they resulted in major trauma to any involved party (including drivers, passengers, and pedestrians). Due to the lack of fault-attribution in the TAD, detailed person-level data was not included in the analysis. The risk factors for major trauma in drunk-driving traffic crashes (i.e., driver-related factors, vehicle-related factors, and environmental factors) were identified via multivariable logistic regression.

**Results:**

We retrieved data from 96,860 drunk-driving traffic crashes during the study period. Registered major trauma from the NHIRD was reported in 9.7% of the crashes. The crash-level driver-related risk factor for major trauma in drunk-driving crashes was heavy drinking (blood alcohol concentration ≥0.55 mg/L; odds ratio = 1.58, 95% confidence interval 1.45–1.72). The vehicle-related risk factors for major trauma in drunk-driving crashes included heavy vehicles, powered two wheelers, and other vehicles. The environmental risk factors for major trauma in drunk-driving crashes included less urbanized regions, late-night crashes, mist, freeways and highways.

**Conclusion:**

Driver-related factors, vehicle-related factors, and environmental factors could all affect the risk of major trauma in drunk driving crashes. A multidisciplinary approach could be taken to further improve the outcomes of drunk driving crashes. The identification of risk factors for trauma could provide guidance for future policy interventions.

## Introduction

The negative effects of alcohol on driving performance can be explained by various mechanisms. For example, alcohol impairs a driver’s divided attention and complex reaction time, which are essential skills for drivers who have to perform multiple operations and process multiple (1). Tracking ability, i.e., the ability to maintain the position of a motor vehicle on a roadway, can also be impaired by alcohol (2). Furthermore, other driving functions, including glare recovery time (3), saccadic and smooth pursuit eye movements (4), peripheral attention (5), and vigilance (6), can be impaired by alcohol, thereby decreasing a driver’s ability to operate motor vehicles.

In addition to driver-related factors, environmental factors also contribute substantially to motor vehicle collisions (MVCs). Previous studies have revealed that factors such as road type, location, weather, and time of day can influence the incidence and severity of MVCs (7–9). When these factors interact with the influence of alcohol, the results might be more detrimental. However, few studies have examined the interactions between environmental factors and driver-related factors and their effects on the severity of injuries in drunk driving crashes. Aside from the basic incidence of drunk-driving crashes, the injury severity caused by these crashes extends the issue of drunk-driving prevention to another dimension. From an individual standpoint, severely injured patients were more susceptible to long-term health sequela, and their ability to return to work and financial income was still affected years after the injury (10, 11). From a community point of view, severe injuries are related to higher medical expenditure, whether in short or long-term (12, 13). Our previous study had demonstrated that sanctions for drunk-driving in Taiwan decreased the incidence of drunk-driving crashes, major trauma related to drunk-driving, and the resulting medical expenditure from Taiwan’s National Health Insurance (NHI) (14), but other factors related to major trauma in drunk-driving crashes had not been analyzed yet. Therefore, the aim of this long-term, nationwide study is to determine how various risk factors affect the severity of injuries in drunk-driving crashes.

## Methods

### Data sources

In this study, data were extracted from the Traffic Accident Data (TAD) provided by the National Police Agency and the National Health Insurance Research Database (NHIRD) provided by the NHI Administration. Both databases are nationwide, population-based, and deidentified. The TAD gathers data from all traffic crashes reported to the police and includes detailed information about each incident, including the time, date, location, individuals involved, breath alcohol concentration (BAC), vehicle type, and road conditions. The data in the NHIRD derive from Taiwan’s NHI, a single-payer system that offers universal coverage to over 99% of the population in Taiwan. Therefore, the NHIRD includes data from all claims related to medical visits, procedures, and prescription medications (15). The data in the TAD and NHIRD could be linked through a 10-digit individual identification (ID) number, allowing us to track individuals involved in traffic crashes and retrieve their corresponding medical information related to the specific event (16). Details of the linkage procedures between the two national databases are described in the Supplementary Document 1.

### Study cohort

We conducted a retrospective observational study of all traffic crashes involving drunk-driving violations that occurred in Taiwan from 2012 to 2019. In our previous study, we discovered that after the law change in December 2011, the trend of drunk-driving and its consequences (hospital admissions, major trauma, medical expenditure, etc.) had reversed from an increasing trend to a decreasing trend ever since (14). In our current study, we focus on analyzing the risk assessment in the era when drunk-driving is in regression, which is more in line with the current drinking behavior. Therefore, 2012 was chosen as the starting point for our analysis. After the Crashes with drunk-driving violations were extracted from the TAD. Drunk-driving was defined as the driver involved in a traffic crash with a BAC above ≥0.15 mg/L, which is compatible with the Road Traffic Management and Penalty Act (17). Crashes with only passengers or pedestrians with an above-legal-limit BAC level were not included in our analysis. By using the individuals’ ID, we found their data in the NHIRD and retrieved their medical records related to the crash. Each crash has a unique process number, which we used to trace and locate all individuals involved in that specific crash. Notably, the TAD does not distinguish between the at-fault driver and the victims, making it impossible to extract the perpetrator’s person-level characteristics, such as age and sex. Records with missing covariate data were excluded (Figure 1).

**Figure 1:**
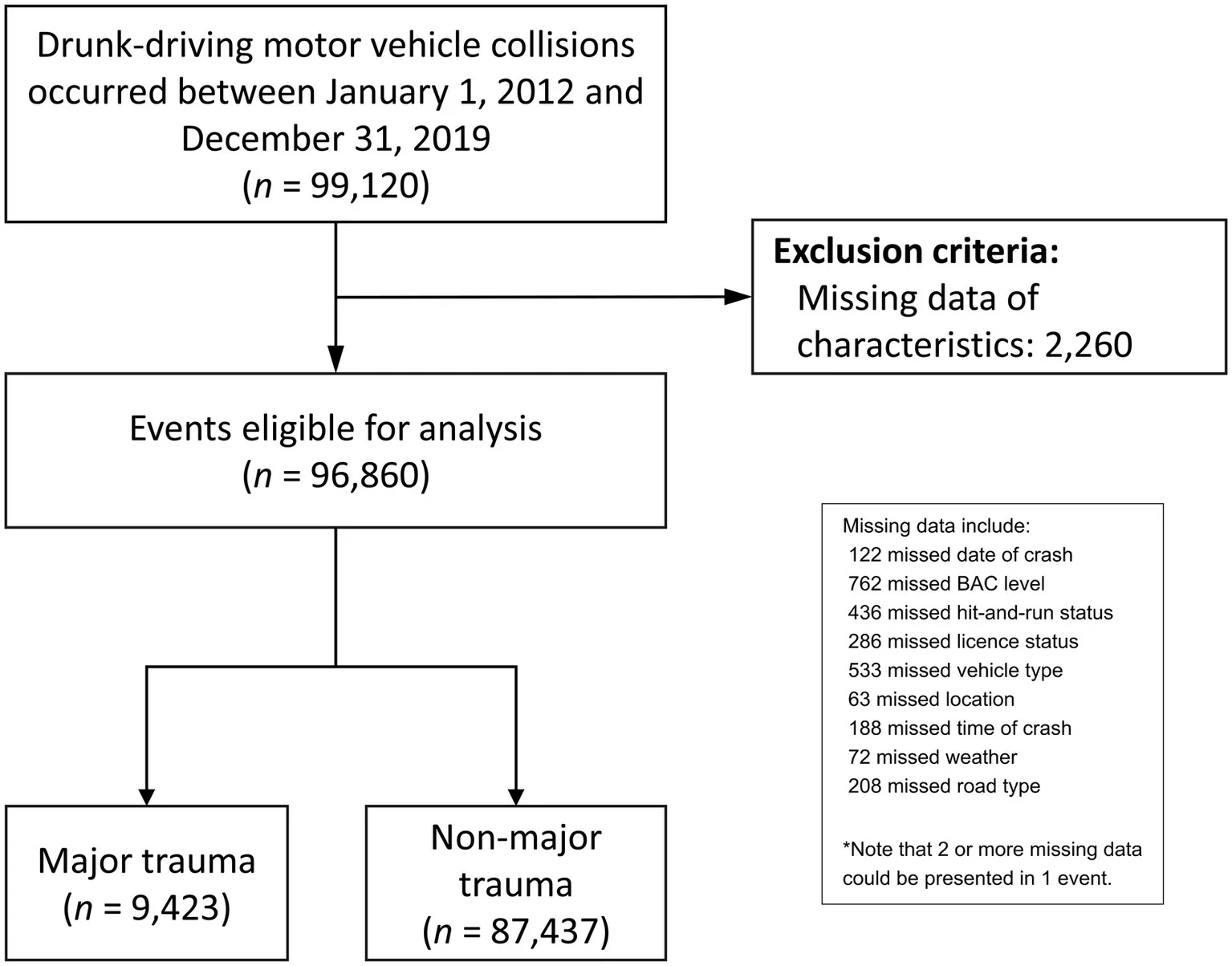
The inclusion and exclusion of drunk-driving motor vehicle collisions in the study.

### Outcome

This study aimed to identify the risk factors for major trauma in drunk-driving MVCs. In this study, major trauma was defined as an injury severity score (ISS) greater than 15 or all-cause death within 60 days after the crash. First proposed by Boyd in 1987, ISS > 15 has long been the most widely accepted definition of major trauma (18, 19). The NHIRD does not include information on the ISS; however, all patients with an ISS of 16 or higher are eligible for a catastrophic illness certificate (CIC), which exempts them from copayments for any medical expenses related to the original trauma. Therefore, the major trauma CIC can serve as a proxy for patients with an ISS ≥ 16. The CIC proxy for major trauma has been a common method to make up for the lack of ISS in trauma-related studies in Taiwan, especially in epidemiologic topics (11, 13, 14, 20–22). Deaths resulting from drunk driving were defined as any patient who died within 60 days after a drunk-driving crash, as recorded in the TAD. We acknowledge that death within 60 days may be sensitive to post-crash care factors and local health system access, rather than reflecting impact forces alone. However, we tend to include as many patients affected by drunk-driving crashes as possible; after all, it is very possible that these patients might not even be in the hospital if it were not for the crash event.

### Risk factors

The potential risk factors were categorized as driver-related factors, vehicle-related factors, and environmental factors. Driver-related factors include BAC, driver’s license eligibility, and hit-and-run status. The BAC was further divided into heavy drinking (BAC ≥ 0.55 mg/L), moderate drinking (BAC 0.25 to <0.55 mg/L), and light drinking (BAC 0.15 to <0.25 mg/L). There are historical reasons for choosing these numbers as the cutoffs for each drunk-driving subgroup. Before June 2013, the threshold for violating administrative drunk-driving rules was 0.25 mg/L, and the threshold for criminal drunk-driving was 0.55 mg/L; after that, the threshold for administrative drunk-driving was lowered to 0.15 mg/L, and the threshold for criminal drunk-driving was 0.25 mg/L (23). Therefore, we chose these numbers to be our cutoff values for each group. The vehicle-related factors were categorized according to the type of vehicle operated by the drunk-driving offender: heavy vehicles, standard vehicles, powered two-wheelers (PTWs), animal/human powered vehicles, and others. Heavy vehicles include trucks, buses, tractors, trailers, or any other vehicles that weigh at least 3,500 kg. Standard vehicles include sedans, wagons, sport utility vehicles (SUVs), vans, pick-up trucks, or other vehicles that weigh less than 3,500 kg. PTW includes motorcycles, scooters, and mopeds. Animal/human-powered vehicles include any transportation vehicle that is powered by humans or animals. The environmental factors included the urbanization level of the MVC location, the time of day during the MVC, the weather, and the road type. The urbanization level is classified according to the standards established by Liu et al. in 2006 (24). All of the above factors could be retrieved from the TAD database.

### Data analysis

All the drunk-driving MVCs during the study period were divided into two groups: major trauma and nonmajor trauma. If an MVC resulted in any related individual suffering from major trauma (ISS ≥ 16) or death, then this MVC was assigned to the major trauma group, whereas MVCs that did not result in major trauma or death were assigned to the nonmajor trauma group. By utilizing the crash-level analysis, this study evaluates the total public health harm generated by a drunk-driving event, rather than isolating individual driver outcomes. However, this event-level specification has distinct analytical implications. First, a crash involving multiple casualties is weighted identically to a crash with a single injured individual. Second, an offender’s measured BAC is modeled against an overall crash outcome, meaning the recorded major trauma or mortality may reflect injury sustained by a passenger, pedestrian, or opposing motorist rather than the intoxicated driver. While this prevents a direct person-level causal analysis of driver self-injury, it aligns with our policy-oriented objective to identify the environmental, vehicle, and driver determinants of severe, community-wide crash outcomes. The temporal trend of the characteristics of drunk-driving MVCs from 2012 to 2019 was tested via a univariate binary logistic regression model (for binary variables) or a multinomial logit model (for categorical variables with ≥3 levels). Driver-related factors, vehicle-related factors, and environmental factors were compared between the two groups. All the factors were categorical; therefore, these factors were analyzed with the chi-square test. Multivariable binary logistic regression models were used to identify the risk factors for major trauma in drunk-driving MVCs. Potential clustering within regions was addressed at the township level (368 townships in Taiwan) by using robust (empirical) standard errors to account for the non-independence of MVCs occurring within the same township. Collinearity was assessed using variance inflation factors (VIFs). All predictors had low VIF values (<2) in the multivariable models, indicating no meaningful multicollinearity. All the statistical analyses were performed with SAS version 9.4 (SAS Institute Inc., Cary, North Carolina, USA). A 2-sided *p* < 0.05 was considered to indicate statistical significance.

### Patient and public involvement

Patients and the public were not involved in this study.

## Results

### Temporal trend of drunk-driving MVCs and major trauma

During the study period, we identified 99,120 drunk-driving MVCs; after excluding records with missing covariate data (2.3%; 2,260/99,120), 96,860 MVCs were eligible for analysis (Figure 1). Major trauma occurred in 9.7% of the MVCs. We analyzed the temporal trends of drunk-driving MVCs and the selected factors over the study period. The number of drunk-driving MVCs exhibited a gradual declining trend, with a reduction from 17,213 drunk-driving MVCs in 2012 to 9,072 incidences in 2019 (Figure 2A). Notably, the overall proportion of major trauma among the drunk-driving MVCs slightly decreased from 10.8% in 2012 to 9.7% in 2019 (*P* trend < 0.001; Figure 2B).

**Figure 2:**
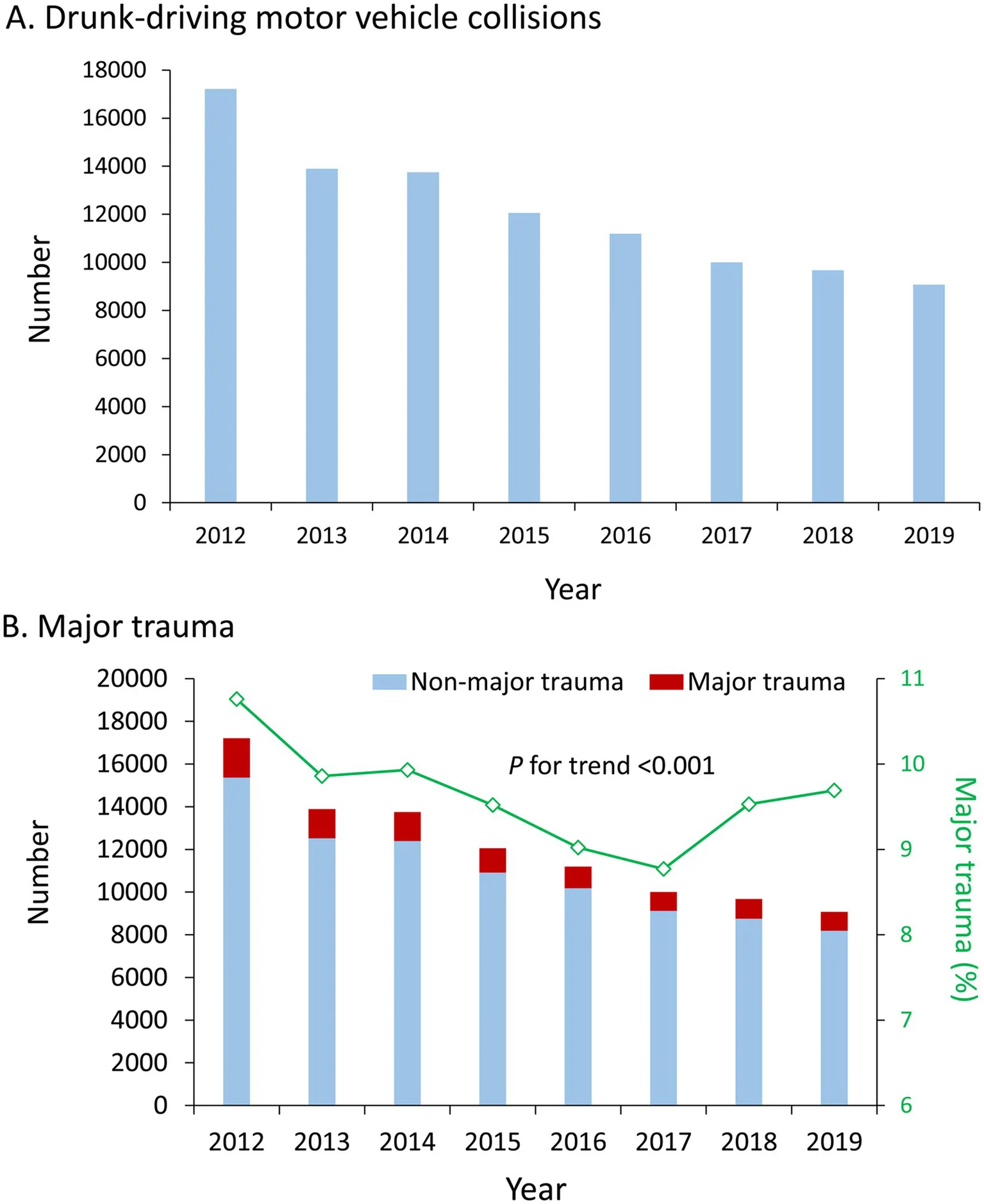
The number of drunk-driving motor vehicle collisions. **(A)** and frequency of major trauma among drunk-driving motor vehicle collisions. **(B)** From 2012 to 2019 in Taiwan.

### Temporal trend of the characteristics of drunk-driving MVCs

Among the driver-related factors, the distribution of the BAC presented an evolving proportion of different BACs. The percentage of heavy drinkers (BAC ≥ 0.55 mg/L) decreased from 67.7% in 2012 to 59.5% in 2019, whereas the percentage of light drinkers (BAC 0.15 to <0.25 mg/L) increased from 7.4 to 12.1% during the same period (*p* trend <0.001; Figure 3A). In contrast, the proportion of drivers without eligible licenses increased from 26.0% in 2012 to 32.4% in 2019 (*p* trend <0.001; Figure 3B). In terms of environmental factors, the number of drunk-driving MVCs in moderately to strongly urbanized regions decreased, whereas the proportion of drunk-driving MVCs in hardly urbanized and nonurbanized regions increased (*p* trend <0.001; Figure 3C). The timing of drunk-driving MVCs also changed during the study period, with the ratio of daytime drunk-driving MVCs (7:00 to 17:59) increasing and the ratio of late-night drunk-driving MVCs (22:00 to 06:59) slowly decreasing (*p* trend <0.001; Figure 3D). Details of the temporal trends in the characteristics of the drunk-driving MVCs are provided in the Supplemental Table 1.

**Figure 3:**
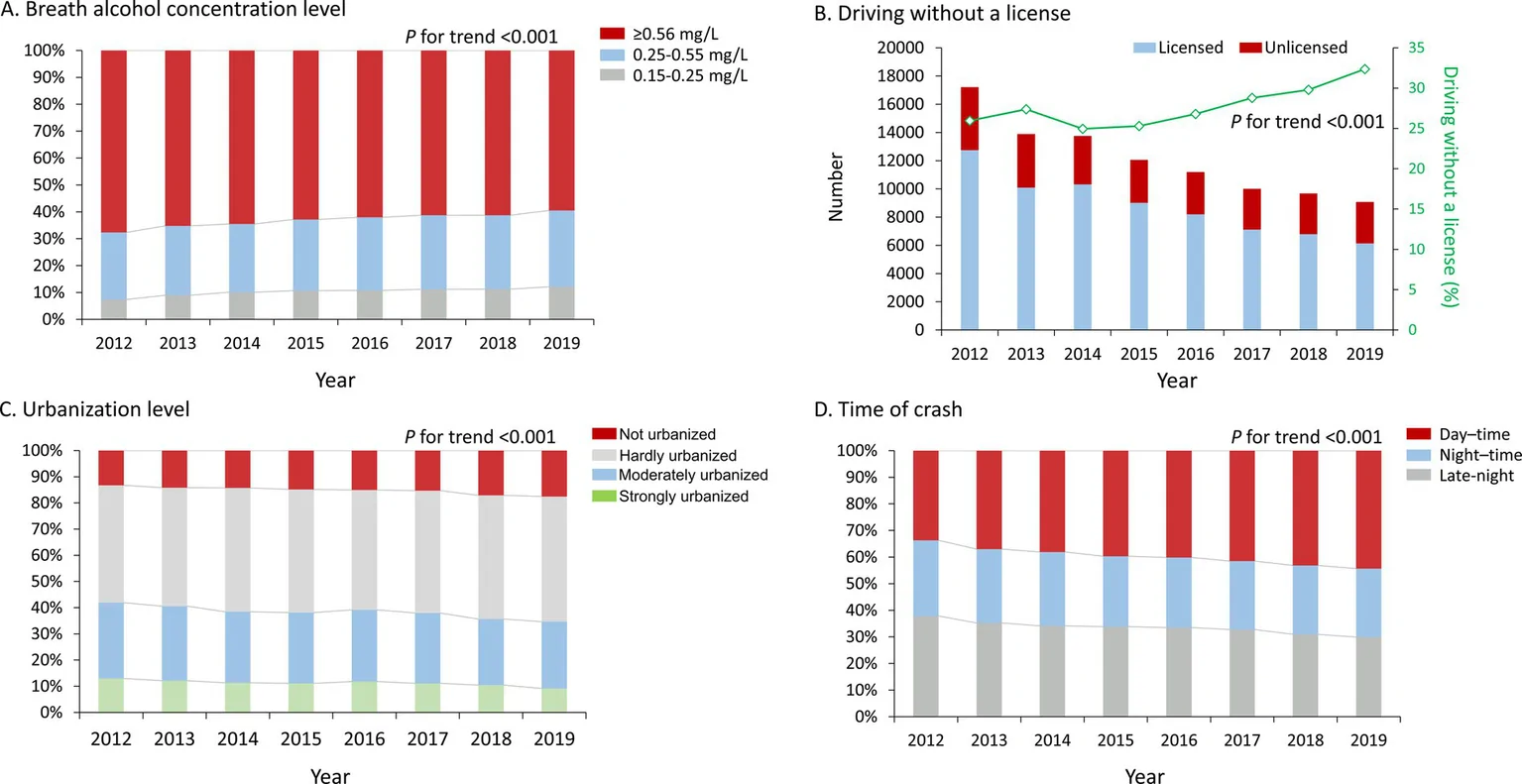
The temporal trend of the blood alcohol concentration for the offender. **(A)**, driving without a license **(B)**, urbanization level of the motor vehicle collisions location **(C)**, and time of crash **(D)** among the drunk-driving motor vehicle collisions from 2012 to 2019 in Taiwan. MVC, motor vehicle collision.

### The characteristics of drunk-driving MVCs with and without major trauma

When the major trauma group was compared with the nonmajor trauma group, several factors were significantly different (see Table 1). In terms of driver-related factors, the major trauma group had a greater proportion of ineligible licenses (29.4% vs. 27.1%, *p* < 0.001) and a lower proportion of light drinkers (BAC 0.15 to <0.25 mg/L) than did the nonmajor trauma group (6.9% vs. 10.3%, *p* < 0.001). The distribution of vehicle-related factors was also significantly different between the two groups, with the major trauma group having more heavy vehicles (0.8% vs. 0.6%) and PTWs (70.4% vs. 64.3%). Among the environmental factors, the distributions of the urbanization of the MVC locations, time of crash, weather, and road types were significantly different between the major trauma group and the nonmajor trauma group (all *p* < 0.001).

**Table 1:** The characteristics of drunk-driving motor vehicle collisions with and without major trauma.

Variable	Total (*n* = 96,860)	Major trauma (*n* = 9,423)	Nonmajor trauma (*n* = 87,437)	*p* value
Driver-related factor				
Breath alcohol concentration				<0.001
0.15 to <0.25 mg/L	9,694 (10.0)	649 (6.9)	9,045 (10.3)	
0.25 to <0.55 mg/L	25,602 (26.4)	1,891 (20.1)	23,711 (27.1)	
≥0.55 mg/L	61,564 (63.6)	6,883 (73.0)	54,681 (62.5)	
Hit and run				0.670
No	94,527 (97.6)	9,190 (97.5)	85,337 (97.6)	
Yes	2,333 (2.4)	233 (2.5)	2,100 (2.4)	
Driving without a license				<0.001
No	70,413 (72.7)	6,656 (70.6)	63,757 (72.9)	
Yes	26,447 (27.3)	2,767 (29.4)	23,680 (27.1)	
Vehicle-related factor				
Drunk-driving vehicle type				<0.001
Heavy vehicles	607 (0.6)	74 (0.8)	533 (0.6)	
Standard vehicles	28,203 (29.1)	2,295 (24.4)	25,908 (29.6)	
Powered two-wheelers	62,816 (64.9)	6,634 (70.4)	56,182 (64.3)	
Animal or human powered vehicles	5,000 (5.2)	389 (4.1)	4,611 (5.3)	
Others	234 (0.2)	31 (0.3)	203 (0.2)	
Environmental factor				
Urbanization level				<0.001
Strongly urbanized	11,068 (11.4)	690 (7.3)	10,378 (11.9)	
Moderately urbanized	26,433 (27.3)	1,889 (20.0)	24,544 (28.1)	
Hardly urbanized	44,889 (46.3)	4,711 (50.0)	40,178 (46.0)	
Not urbanized	14,470 (14.9)	2,133 (22.6)	12,337 (14.1)	
Time of crash				<0.001
Day–time (0700–1759)	37,808 (39.0)	3,384 (35.9)	34,424 (39.4)	
Night–time (1800–2,159)	26,142 (27.0)	2,454 (26.0)	23,688 (27.1)	
Late–night (2200–0659)	32,910 (34.0)	3,585 (38.0)	29,325 (33.5)	
Weather				<0.001
Equable	86,601 (89.4)	8,435 (89.5)	78,166 (89.4)	
Rain	10,114 (10.4)	959 (10.2)	9,155 (10.5)	
Mist	98 (0.1)	20 (0.2)	78 (0.1)	
Gale	47 (0.1)	9 (0.1)	38 (0.0)	
Road types				<0.001
General roads	88,337 (91.2)	8,230 (87.3)	80,107 (91.6)	
Freeway	703 (0.7)	107 (1.1)	596 (0.7)	
Highway	4,874 (5.0)	763 (8.1)	4,111 (4.7)	
Others	2,946 (3.0)	323 (3.4)	2,623 (3.0)	

Data are presented as frequency and percentage.

### Risk factors for major trauma in drunk-driving MVCs

Table 2 summarizes the risk factors for major trauma in drunk-driving MVCs. In terms of driver-related factors, only heavy drinking (BAC ≥ 0.55 mg/L) was an independent risk factor for major trauma (odds ratio [OR] = 1.58, 95% confidence interval [CI] 1.45–1.72). Among the vehicle-related factors, heavy vehicles (OR = 1.74, 95% CI 1.36–2.24), PTWs (OR = 1.34, 95% CI 1.28–1.42), and other vehicles (OR = 1.80, 95% CI 1.23–2.65) were risk factors for major trauma. There were several environmental risk factors for major trauma, including moderately urbanized regions (OR = 1.16, 95% CI 1.06–1.27), hardly urbanized regions (OR = 1.75, 95% CI 1.61–1.90), and not urbanized regions (OR = 2.47, 95% CI 2.25–2.71). Regarding the time of the MVC, late-night MVCs (OR = 1.29, 95% CI 1.23–1.36) constituted a risk factor for major trauma. Regarding weather conditions, mist (OR = 1.91, 95% CI 1.16–3.15) was significantly associated with major trauma, whereas rain and wind were not significantly associated with major trauma. However, the frequencies of mist and gale in our total sample of weather factors are rather low. Regarding road types, freeways (OR = 1.92, 95% CI 1.55–2.37) and highways (OR = 1.43, 95% CI 1.31–1.55) were both associated with major trauma in drunk-driving crashes.

**Table 2:** Factors associated with any major traumas in drunk-driving motor vehicle collisions.

Variable	Univariate logistic regression		Multivariable logistic regression	
	Odds ratio (95% CI)	*p* value	Odds ratio (95% CI)	*p* value
Driver-related factor				
Breath alcohol concentration				
0.15 to <0.25 mg/L	Reference		Reference	
0.25 to <0.55 mg/L	1.11 (1.01–1.22)	0.025	1.08 (0.99–1.19)	0.092
≥0.55 mg/L	1.75 (1.61–1.91)	<0.001	1.58 (1.45–1.72)	<0.001
Hit and run	1.03 (0.90–1.18)	0.666	1.14 (0.99–1.31)	0.064
Driving without a license	1.12 (1.07–1.17)	<0.001	1.00 (0.95–1.05)	0.919
Vehicle-related factor				
Drunk-driving vehicle type				
Heavy vehicles	1.57 (1.23–2.01)	<0.001	1.74 (1.36–2.24)	<0.001
Standard vehicles	Reference		Reference	
Powered two-wheelers	1.33 (1.27–1.40)	<0.001	1.34 (1.28–1.42)	<0.001
Animal or human powered vehicles	0.95 (0.85–1.07)	0.394	0.99 (0.88–1.11)	0.842
Others	1.73 (1.18–2.52)	0.005	1.80 (1.23–2.65)	0.003
Environmental factor				
Urbanization level				
Strongly urbanized	Reference		Reference	
Moderately urbanized	1.16 (1.06–1.27)	0.002	1.16 (1.06–1.27)	0.001
Hardly urbanized	1.76 (1.62–1.92)	<0.001	1.75 (1.61–1.90)	<0.001
Not urbanized	2.60 (2.38–2.85)	<0.001	2.47 (2.25–2.71)	<0.001
Time of crash				
Daytime (0,700–1,759)	Reference		Reference	
Night–time (1,800–2,159)	1.05 (1.00–1.11)	0.060	1.01 (0.96–1.07)	0.622
Late–night (2,200–0,659)	1.24 (1.18–1.31)	<0.001	1.29 (1.23–1.36)	<0.001
Weather				
Equable	Reference		Reference	
Rain	0.97 (0.91–1.04)	0.407	0.98 (0.91–1.05)	0.622
Mist	2.38 (1.45–3.89)	<0.001	1.91 (1.16–3.15)	0.011
Gale	2.20 (1.06–4.54)	0.034	1.85 (0.89–3.85)	0.102
Road types				
General roads	Reference		Reference	
Freeway	1.75 (1.42–2.15)	<0.001	1.92 (1.55–2.37)	<0.001
Highway	1.81 (1.67–1.96)	<0.001	1.43 (1.31–1.55)	<0.001
Others	1.20 (1.07–1.35)	0.003	1.08 (0.96–1.22)	0.186

CI, confidence interval.

## Discussion

Road traffic-related injuries have long been a global issue. Since 2000, the number of annual road traffic deaths worldwide has exceeded 1 million (25), and a large proportion of these fatalities are alcohol related. According to the World Health Organization, approximately 20% of fatally injured drivers have BACs above the legal limit in high-income countries. Studies also reported that between 33 and 69% of fatally injured drivers in middle- and low-income countries consumed alcohol before their crashes (26). In addition to preventing drivers from committing drunk driving, a problem-oriented harm reduction approach can also be helpful in decreasing the probability of road injuries (27). For example, the Data Driven Approaches to Crime and Traffic Safety program in Kansas, United States, has shown that hot-spot policing can reduce traffic collisions with injuries (28). In this study, we followed a similar process. By identifying risk factors for drunk driving crashes, we can make smarter adjustments for preventive measures in the future.

Regarding driver-related factors, we found that heavy drinking (BAC ≥ 0.55 mg/L) significantly increased the likelihood of a crash resulting in a major trauma outcome for at least one involved individual. Alcohol clearly impairs a driver’s physical performance via various mechanisms (29). The detrimental effects of alcohol are dose-dependent (30), and some researchers have even suggested that even a BAC of 0.01% is significantly more dangerous than a BAC of 0% (31). Since our dataset included only drunk-driving crashes, the risk for those who ingested alcohol but did not surpass the legal BAC limit was not evaluated. In addition, moderate drinking (0.25 to <0.55 mg/L) was associated with a marginally increased risk of major trauma, which echoed previous studies reporting that the detrimental effects of alcohol are dose-dependent. During our study period, the BAC limit decreased from the original 0.25 mg/L to 0.15 mg/L in 2013 (23), and our results may partly support the adjustment, from a retrospective observational perspective.

With respect to vehicle-related factors, we found that drunk-driving crashes involving heavy vehicles and PTWs carried higher odds of resulting in severe or fatal event-level outcomes. Previous studies have shown that heavy vehicles are associated with greater injury severity (32); therefore, it is not surprising that the same risk exists when alcohol is involved. PTWs are another important issue in Taiwan. Taiwan is one of the countries with the highest prevalence of PTWs in the world (33); therefore, these vehicles pose a special threat to road safety. Owing to the limited protection offered by PTWs, injuries to such vehicles can be unpredictable, and polytrauma is very common (34). These injuries inevitably result in higher mortality rates compared with those of cars (35). Alcohol intoxication is even more detrimental for PTW drivers, since these drivers are more likely to lose control and balance of their vehicles, and the rate of helmet use decreases among intoxicated individuals (36). In Taiwan, PTW-targeted policies are not uncommon. For example, mandatory helmet use for PTW drivers and passengers has had outstanding effects on preventing head injuries (37). Therefore, an alcohol prevention program aimed at PTWs and heavy vehicles could be a potential policy intervention in the future.

We observed that the risk for major trauma after drunk driving was inversely proportional to the level of urbanization. The cause of this phenomenon can be multifactorial. First, nonurban areas in Taiwan are generally more short-staffed and are less efficient when handling traffic affairs (38). A shortage of manpower could lead to a decrease in law enforcement intensity, which is pivotal for deterring drunk-driving behaviors (39). Additionally, the accessibility of both public transportation and designated driver services is lower in nonurban areas in Taiwan (40, 41), and this discrepancy might cause drivers to take risks, even after heavy drinking. Inequality in medical infrastructure might also be a confounding factor. The medical resources in urban areas considerably exceed those in rural townships in Taiwan, and this discrepancy has a considerable impact on trauma care (20). Theoretically, injury severity is determined at the time of the crash, and subsequent trauma care should not change the severity. However, according to our study design, major trauma patients included patients with an ISS ≥ 16 and those who died in or after a drunk-driving crash. It is possible that some patients with an ISS lower than 16 died due to inferior medical resources in rural areas, thereby increasing the total number of major traumas in our cohort. Therefore, the solution to problems in nonurban areas should also be a multisectoral approach that includes law enforcement, public transport, and medical domains.

Several other environmental factors, including late-night, mist, freeways, and highways, were found to be associated with a greater risk of major trauma. These factors are largely nonmodifiable, but some approaches might be helpful to ameliorate these environmental risks. For example, foggy weather is dangerous because of its adverse effects on visibility (42). Therefore, road lighting improvements, highly visible warning signs before fog-prone areas, increased police visibility, and real-time fog warning systems are all measures that the government could take to lower the risk for all road users (43, 44). However, we do have to acknowledge the relatively low frequency of mist under enlisted weather conditions, and the confidence level in our presented results was wide, indicating that the true impact of mist on Taiwan’s drunk-driving should be further investigated. Also, the operational definitions of each weather condition in TAD are not clearly specified, so ambiguity may affect our results. Late-night crashes are another issue. Similar to weather conditions, one cannot change the time of day, but by identifying vulnerable groups that are prone to late-night traffic crashes, we can lower the risk exposure of susceptible groups and conduct tailored prevention programs. For example, Houwing and Twisk reported that young males in the Netherlands were prone to late-night traffic injuries and were often exposed to alcohol. Therefore, specified preventive measures, such as designated driver programs, increased law enforcement intensity, and education, could be the next step for policy intervention (45). In addition to the possible improvements mentioned before, better highway and freeway designs could also help ameliorate the “black spot” of traffic crashes (46) and thus decrease drunk-driving-related major trauma.

There are some noteworthy trends in our results. The annual number of drunk driving events and the related number of major traumas both showed a gradual declining trend, indicating that preventing drunk driving events within our study period was effective. The proportion of heavy drinkers is also decreasing, which may be the result of the policy intervention in 2013, which lowered the BAC limit to 0.15 mg/L (47). The proportion of drivers without eligible licenses is increasing. However, this phenomenon is not limited to drunk-driving offenders; rather, it is a nationwide problem among all drivers (48). Despite the declining trend of overall drunk-driving, the number of daytime drunk-driving violations has increased. The Ministry of Transport postulated that this could be due to neglecting the “hangover” effect from the previous night’s drinking (49). With respect to the upward trend in drunk driving in less urbanized areas, the underlying causes cannot be definitively established using our current dataset. One plausible, though unverified, interpretation is a potential disparity in regional law enforcement intensity. It is conceivable that urban police departments scaled up targeted anti-drunk-driving operations or checkpoint frequencies more rapidly during this timeframe, while nonurban departments may have faced resource constraints that slowed comparable enforcement adoption. However, because our database lacks localized data on police resource allocation, checkpoint deployments, or enforcement metrics, this mechanism remains entirely hypothetical and requires future empirical validation.

There are several limitations in this study. A potential source of bias is the calculation of major trauma. As mentioned in the methodology section, data from major trauma patients were extracted based on the CIC. The application for CICs is sent by physicians and hospitals. In some cases, physicians might not apply for a CIC for patients with major trauma. Therefore, the exact volume of major trauma patients could be underestimated by our patient selection method (22). However, using the CIC as a proxy for major trauma is our only means in the NHIRD because the ISS score is not documented in the NHIRD, and this proxy is used throughout the entire study period. Considering that our analysis focused on the yearly trend of major trauma, this bias should affect each year equally; hence, the overall trend should not be compromised theoretically.

Another major limitation lies within our crash-level analysis. Because our outcome is event-based rather than person-based, our models do not differentiate between injuries to the drunk driver and to innocent third parties, nor do they account for the total number of individuals injured per crash. While appropriate for assessing broad public health and policy impacts, future studies using person-level data linkage are required to cleanly isolate individual offender-to-victim injury pathways and model cumulative passenger-load risks. It is also worth noting that some potentially important confounding factors may have been missed in our analysis, e.g., sex, age, and vehicle speed. The main reason these factors could not be well analyzed is that the TAD did not include fault attribution for each crash, so the interaction between factors could not be easily clarified. For instance, if we conclude that a certain sex or age group was statistically associated with increased risk on the road without attributing fault, it would be more confusing for readers to interpret our results.

Nevertheless, another bias could arise from changes in sanctions during our study period, which might affect case mix in our analysis. However, the drunk-driving trend from 2012 to 2019 is downward, compared to the era before 2012. Therefore, the analysis is still based on the same declining era of drunk driving. Additionally, drinking behavior, road culture, and weather can vary dramatically across regions. Therefore, the risk factors for major trauma identified in our study may not be generalizable to other countries. Finally, although the excluded missing data accounted for only a small proportion of the cohort (2.3%; 2,260/99,120), such exclusions could still introduce differential bias and may affect the internal validity of our findings to some extent.

## Conclusion

From 2012 to 2019, the number of drunk-driving crashes in Taiwan and the prevalence of major trauma in drunk-driving crashes declined steadily. We have identified several robust risk factors associated with major trauma in drunk-driving crashes from the crash level. These factors included: heavy drinking, heavy vehicles, PTWs, high-speed roads (freeways and highways), less urbanized regions (moderately/hardly/not urbanized compared to strongly urbanized), and late-night crashes. The driver-related risk factor for major trauma was heavy drinking. Vehicle-related risk factors for major trauma included heavy vehicles, PTWs, and other vehicles. Since these risk factors span across individual, vehicle, and environmental domains, the findings of this study could guide future policy interventions, especially in a multidisciplinary approach.

## Data Availability

The datasets presented in this article are not readily available because the datasets generated and/or analyzed during the current study are not publicly available owing to patient confidentiality restrictions by the NHIRD, but are available from the NHIRD upon reasonable request. Requests to access the datasets should be directed to st0221@mohw.gov.tw.
