# Supplementary material for: Driver, vehicle, and environmental risk factors for crash-level major trauma in drunk driving collisions: a national registry linkage study
Source: Front Public Health. 2026 Sep 28;14:1733604. doi: 10.3389/fpubh.2026.1733604 (PMC13655238; doi:10.3389/fpubh.2026.1733604)
Supplement: Supplementary file 1 [file Supplementary_file_1.docx]

Supplemental document 1:

Data Linkage, Encryption, and Privacy Protection Protocols

1. Data Linkage Infrastructure

To evaluate post-accident clinical courses and healthcare utilization, individual-level data linkage was executed between the Traffic Accident Data (TAD)—compiled and maintained by the National Police Agency—and the National Health Insurance Research Database (NHIRD), administered by the National Health Insurance Administration. This secure cross-registry integration was performed exclusively within the controlled physical and virtual environment of the Health and Welfare Data Science Center (HWDC) of the Ministry of Health and Welfare in Taiwan.

2. De-identification and Cryptographic Protocols

To ensure absolute patient confidentiality and eliminate the risk of identity disclosure, all records underwent a rigorous, multi-layered de-identification process within the HWDC prior to analysis. Personal identification numbers and sensitive identifiers were permanently converted into non-identifiable, hashed research IDs. To prevent cross-project re-identification or unauthorized data reconstruction, this cryptographic process was strictly applicant-specific. The unique hashing keys assigned to this project differed from those allocated to other research initiatives, thereby prohibiting the cross-linking of datasets across unauthorized research boundaries.

3. Regulatory and Legal Compliance

The data governance, security, and anonymization workflows deployed in this study strictly complied with Taiwan’s National Standards for information security and privacy protection. Specifically, the de-identification practices adhered to CNS 29100 (Privacy Framework) and CNS 29191 (Requirements for De-identification), both of which are harmonized with international information security frameworks. Under Taiwan’s Personal Data Protection Act (PDPA), datasets that have been irreversibly de-identified and anonymized such that individuals can no longer be directly or indirectly identified are formally designated as outside the restricted scope of personal data. Consequently, the analytical dataset utilized in this study fully satisfies all ethical, legal, and institutional data-sharing mandates.

4. Linkage Integrity and Error Mitigation

Prior to institutional encryption and anonymization within the HWDC, individual records in both the TAD and NHIRD databases were linked deterministically. This linkage utilized the universally mandated national identification number as the unique primary key. Because the matching algorithm relied on this single, verified national identifier before the subsequent cryptographic transformation of research IDs, the potential for mismatching, registration bias, or cross-registry linkage errors was considered negligible, securing high data integrity for the longitudinal cohort.
