# Supplementary material for: Driver, vehicle, and environmental risk factors for crash-level major trauma in drunk driving collisions: a national registry linkage study
Source: Front Public Health. 2026 Sep 28;14:1733604. doi: 10.3389/fpubh.2026.1733604 (PMC13655238; doi:10.3389/fpubh.2026.1733604)
Supplement: Supplementary file 2 [file Table_1.docx]

**Supplemental Table 1**. The temporal trend of the characteristics of drunk-driving motor vehicle collisions

| Variable | Total  (*n* = 96,860) | 2012  (*n* = 17,213) | 2013  (*n* = 13,893) | 2014  (*n* = 13,753) | 2015  (*n* = 12,059) | 2016  (*n* = 11,196) | 2017  (*n* = 10,000) | 2018  (*n* = 9,674) | 2019  (*n* = 9,072) | *P* for  trend |
| --- | --- | --- | --- | --- | --- | --- | --- | --- | --- | --- |
| **Major trauma** | 9,423 (9.7) | 1,852 (10.8) | 1,370 (9.9) | 1,365 (9.9) | 1,148 (9.5) | 1,010 (9.0) | 877 (8.8) | 922 (9.5) | 879 (9.7) | <0.001 |
| **Driver-related factor** |  |  |  |  |  |  |  |  |  |  |
| Breath alcohol concentration |  |  |  |  |  |  |  |  |  | <0.001 |
| 0.15–0.25 mg/L | 9,694 (10.0) | 1,272 (7.4) | 1,243 (9.0) | 1,388 (10.1) | 1,286 (10.7) | 1,204 (10.8) | 1,118 (11.2) | 1,085 (11.2) | 1,098 (12.1) |  |
| 0.25–0.55 mg/L | 25,602 (26.4) | 4,297 (25.0) | 3,583 (25.8) | 3,492 (25.4) | 3,195 (26.5) | 3,045 (27.2) | 2,755 (27.6) | 2,660 (27.5) | 2,575 (28.4) |  |
| >0.55 mg/L | 61,564 (63.6) | 11,644 (67.7) | 9,067 (65.3) | 8,873 (64.5) | 7,578 (62.8) | 6,947 (62.1) | 6,127 (61.3) | 5,929 (61.3) | 5,399 (59.5) |  |
| Hit and run | 2,333 (2.4) | 440 (2.6) | 346 (2.5) | 299 (2.2) | 300 (2.5) | 282 (2.5) | 239 (2.4) | 221 (2.3) | 206 (2.3) | 0.187 |
| Driving without a license | 26,447 (27.3) | 4,466 (26.0) | 3,803 (27.4) | 3,432 (25.0) | 3,051 (25.3) | 2,999 (26.8) | 2,878 (28.8) | 2,882 (29.8) | 2,936 (32.4) | <0.001 |
| **Vehicle-related factor** |  |  |  |  |  |  |  |  |  |  |
| Drunk-driving vehicle type |  |  |  |  |  |  |  |  |  | <0.001 |
| Heavy vehicles | 607 (0.6) | 90 (0.5) | 102 (0.7) | 96 (0.7) | 76 (0.6) | 76 (0.7) | 57 (0.6) | 54 (0.6) | 56 (0.6) |  |
| Standard vehicles | 28,203 (29.1) | 4,965 (28.8) | 3,768 (27.1) | 3,886 (28.3) | 3,549 (29.4) | 3,508 (31.3) | 3,093 (30.9) | 2,817 (29.1) | 2,617 (28.9) |  |
| Powered two-wheelers | 62,816 (64.9) | 11,781 (68.4) | 9,571 (68.9) | 9,152 (66.6) | 7,754 (64.3) | 6,899 (61.6) | 6,154 (61.5) | 6,007 (62.1) | 5,498 (60.6) |  |
| Animal/ human powered vehicles | 5,000 (5.2) | 336 (2.0) | 413 (3.0) | 594 (4.3) | 653 (5.4) | 682 (6.1) | 675 (6.8) | 771 (8.0) | 876 (9.7) |  |
| Others | 234 (0.2) | 41 (0.2) | 39 (0.3) | 25 (0.2) | 27 (0.2) | 31 (0.3) | 21 (0.2) | 25 (0.3) | 25 (0.3) |  |
| **Environmental factor** |  |  |  |  |  |  |  |  |  |  |
| Urbanization level |  |  |  |  |  |  |  |  |  | <0.001 |
| Strongly urbanized | 11,068 (11.4) | 2,229 (13.0) | 1,681 (12.1) | 1,556 (11.3) | 1,337 (11.1) | 1,319 (11.8) | 1,110 (11.1) | 1,006 (10.4) | 830 (9.2) |  |
| Moderately urbanized | 26,433 (27.3) | 4,982 (28.9) | 3,959 (28.5) | 3,730 (27.1) | 3,258 (27.0) | 3,063 (27.4) | 2,687 (26.9) | 2,441 (25.2) | 2,313 (25.5) |  |
| Hardly urbanized | 44,889 (46.3) | 7,714 (44.8) | 6,285 (45.2) | 6,504 (47.3) | 5,676 (47.1) | 5,130 (45.8) | 4,672 (46.7) | 4,572 (47.3) | 4,336 (47.8) |  |
| Not urbanized | 14,470 (14.9) | 2,288 (13.3) | 1,968 (14.2) | 1,963 (14.3) | 1,788 (14.8) | 1,684 (15.0) | 1,531 (15.3) | 1,655 (17.1) | 1,593 (17.6) |  |
| Time of crash |  |  |  |  |  |  |  |  |  | <0.001 |
| Daytime (0700–1759) | 37,808 (39.0) | 5,803 (33.7) | 5,142 (37.0) | 5,238 (38.1) | 4,788 (39.7) | 4,493 (40.1) | 4,149 (41.5) | 4,172 (43.1) | 4,023 (44.4) |  |
| Night–time (1800–2159) | 26,142 (27.0) | 4,898 (28.5) | 3,860 (27.8) | 3,818 (27.8) | 3,192 (26.5) | 2,953 (26.4) | 2,574 (25.7) | 2,510 (26.0) | 2,337 (25.8) |  |
| Late–night (2200–0659) | 32,910 (34.0) | 6,512 (37.8) | 4,891 (35.2) | 4,697 (34.2) | 4,079 (33.8) | 3,750 (33.5) | 3,277 (32.8) | 2,992 (30.9) | 2,712 (29.9) |  |
| Weather |  |  |  |  |  |  |  |  |  | 0.055 |
| Equable | 86,601 (89.4) | 15,082 (87.6) | 12,490 (89.9) | 12,598 (91.6) | 11,087 (91.9) | 9,637 (86.1) | 8,966 (89.7) | 8,619 (89.1) | 8,122 (89.5) |  |
| Rain | 10,114 (10.4) | 2,092 (12.2) | 1,384 (10.0) | 1,136 (8.3) | 961 (8.0) | 1,540 (13.8) | 1,022 (10.2) | 1,038 (10.7) | 941 (10.4) |  |
| Mist | 98 (0.1) | 33 (0.2) | 15 (0.1) | 13 (0.1) | 3 (0.02) | 10 (0.1) | 4 (0.04) | 13 (0.1) | 7 (0.1) |  |
| Gale | 47 (0.1) | 6 (0.03) | 4 (0.03) | 6 (0.04) | 8 (0.1) | 9 (0.1) | 8 (0.1) | 4 (0.04) | 2 (0.02) |  |
| Road types |  |  |  |  |  |  |  |  |  | <0.001 |
| General roads | 88,337 (91.2) | 15,692 (91.2) | 12,703 (91.4) | 12,642 (91.9) | 10,952 (90.8) | 10,193 (91.0) | 9,132 (91.3) | 8,755 (90.5) | 8,268 (91.1) |  |
| Freeway | 703 (0.7) | 101 (0.6) | 90 (0.7) | 77 (0.6) | 110 (0.9) | 90 (0.8) | 88 (0.9) | 70 (0.7) | 77 (0.9) |  |
| Highway | 4,874 (5.0) | 1,018 (5.9) | 740 (5.3) | 651 (4.7) | 598 (5.0) | 561 (5.0) | 439 (4.4) | 484 (5.0) | 383 (4.2) |  |
| Others | 2,946 (3.0) | 402 (2.3) | 360 (2.6) | 383 (2.8) | 399 (3.3) | 352 (3.1) | 341 (3.4) | 365 (3.8) | 344 (3.8) |  |

Data are presented as frequency and percentage.
